# Biomarkers of Radiotherapy-Induced Immunogenic Cell Death

**DOI:** 10.3390/cells10040930

**Published:** 2021-04-17

**Authors:** Rianne D. W. Vaes, Lizza E. L. Hendriks, Marc Vooijs, Dirk De Ruysscher

**Affiliations:** 1 Department of Radiation Oncology (MAASTRO), GROW School for Oncology and Developmental Biology, Maastricht University Medical Center, P.O. 616, 6200 MD Maastricht, The Netherlands; marc.vooijs@maastrichtuniversity.nl (M.V.); dirk.deruysscher@maastro.nl (D.D.R.); 2 Department of Pulmonary Diseases, GROW School for Oncology and Developmental Biology, Maastricht University Medical Center, P.O. 616, 6200 MD Maastricht, The Netherlands; lizza.hendriks@mumc.nl

**Keywords:** immunogenic cell death, radiotherapy, biomarkers, HMGB1, calreticulin, Hsp70, interferon, necroptosis

## Abstract

Radiation therapy (RT) can induce an immunogenic variant of regulated cell death that can initiate clinically relevant tumor-targeting immune responses. Immunogenic cell death (ICD) is accompanied by the exposure and release of damage-associated molecular patterns (DAMPs), chemokine release, and stimulation of type I interferon (IFN-I) responses. In recent years, intensive research has unraveled major mechanistic aspects of RT-induced ICD and has resulted in the identification of immunogenic factors that are released by irradiated tumor cells. However, so far, only a limited number of studies have searched for potential biomarkers that can be used to predict if irradiated tumor cells undergo ICD that can elicit an effective immunogenic anti-tumor response. In this article, we summarize the available literature on potential biomarkers of RT-induced ICD that have been evaluated in cancer patients. Additionally, we discuss the clinical relevance of these findings and important aspects that should be considered in future studies.

## 1. Introduction

Radiation therapy (RT) is a treatment modality used in over 50% of all cancer patients, either alone or more commonly in combination with surgery and/or chemotherapy [1,2]. Since the discovery of X-rays by Röntgen in 1895 [3], in the past decade, the field of RT has undergone a paradigm shift. For years, the widespread view was that ionizing irradiation does only act locally on tumor cells by inducing DNA double-strand breaks followed by some form of regulated cell death, including apoptosis, necrosis, autophagy, mitotic catastrophe, or replicative senescence [4]. For that reason, research aiming to improve RT outcomes mainly focused on the cancer cell itself, ignoring the complex biological interaction between the tumor and the neighboring cells that are present in the tumor microenvironment (TME). Consequently, classical radiobiology largely failed to appreciate the effects of RT on the TME and the subsequent systemic immunological alterations, although the responses that are triggered in the TME may be critical in determining the efficacy of the treatment regimen.

To date, it has been well-established that RT can also induce clinically relevant tumor-targeting immune responses, which critically rely on the antigenicity of cancer cells and their capacity to generate adjuvant signals [5]. In particular, RT-induced immunogenic cell death (ICD), which is defined as ‘a form of regulated cell death that is sufficient to activate an adaptive immune response in immunocompetent hosts’ [6], is known to contribute to the propagation of antitumor immunity [4,7,8]. Key events that can result in the activation of RT-induced ICD signaling include the induction of DNA damage, the generation of reactive oxygen species, and the induction of a stress response in subcellular organelles, such as endoplasmic reticulum (ER) and mitochondria [9]. DNA normally resides in the nucleus and mitochondria, however, RT-induced DNA damage can result in the accumulation of DNA in the cytoplasm. Its presence in the cytoplasm serves as a danger-associated molecular pattern (DAMP) to trigger an immune response [10]. Cyclic guanosine monophosphate (GMP)-adenosine monophosphate (AMP) synthase (cGAS) is the sensor that detects DNA as a DAMP, which activates the downstream adaptor molecule stimulator of interferon genes (STING) [11]. Activation of the cGAS-STING pathway stimulates the expression of type I interferon (IFN-I) that can initiate an IFN-I driven inflammatory program [12,13,14]. Furthermore, the combined action of reactive oxygen species and ER stress underlies the activation of additional danger signaling pathways that include the translocation and release of DAMPs, chemokine release, and stimulation of IFN-I responses [9]. The RT-induced immunogenic response results in the uptake of tumor antigens by dendritic cells (DCs) that present these tumor antigens in the tumor-draining lymph nodes to cytotoxic T lymphocytes to prime and activate anti-tumor immunity [15,16]. In addition to these local immunological effects, preclinical models as well as clinical observations revealed that loco-regionally delivered RT can also trigger tumor immune responses outside the radiation field, called the abscopal effect [17,18,19]. However, these effects are often weak, therefore, it is suggested that combination with immunotherapies may induce stronger adaptive immune responses [20,21].

Despite major improvements in the treatment of cancer patients with immunotherapy, primary therapy resistance, defined as a lack of clinical benefit from immunotherapy on tumor growth, exists in a large proportion of patients. On top of this, and maybe even more important, a substantial percentage of tumors show progression after an initial response [22,23]. In the clinical setting, not much is known regarding the identification of patients that will benefit from RT treatment combined with immunotherapy, or resistance to immunotherapy. The development of novel (immunological) treatment strategies for the optimal priming of the immune system is hampered by the lack of biomarkers for monitoring the treatment response and the selection of responsive patients and the most effective treatment combinations. This highlights the need for clear biomarkers that can be used to define synergistic treatment combinations. Although several immunogenic factors have been shown to be released by irradiated tumor cells, so far, only a limited number of studies searched for potential predictive immunological biomarkers in the clinical setting. The aim of this review is to summarize and discuss the current knowledge on biomarkers of RT-induced ICD in cancer patients.

## 2. Potential Biomarkers of RT-Induced ICD in Cancer Patients

In recent years, intensive research has unraveled major mechanistic aspects of ICD that contribute to the activation of adaptive immunity against dying cancer cells [24]. In particular, preclinical studies have shown that exposure of cancer cells to potential ICD inducers results in the expression and release of numerous DAMPS and cytokines that have been mechanistically linked to the initiation of an adaptive immune response. These immunostimulatory molecules include ATP [25,26], cellular nucleic acids [27,28,29], high mobility group box 1 (HMGB1) [30], ANXA1 [31], cytokines like IFN-I, CCL2, CXCL1, and CXCL10 [28,32,33], and ER chaperones like calreticulin, ERp57, Hsp70, Hsp90 [34,35,36]. These ICD-associated DAMPs and cytokines are known 1) to enable the recruitment, maturation, and cross-presentation activity of antigen-presenting cells, 2) to promote the uptake of dying cells by antigen-presenting cells, and 3) to promote T-cell and neutrophil recruitment.

Currently, no reliable biomarkers are available that can be used to predict if irradiated tumor cells undergo ICD that can elicit an effective immunogenic anti-tumor response. Table 1 summarizes potential biomarkers of RT-induced ICD that have been evaluated in cancer patients. These potential biomarkers are also discussed in more detail below.

### 2.1. DAMPs

#### 2.1.1. Calreticulin

RT-induced ER stress is accompanied by the accumulation of misfolded and unfolded protein in the lumen of the ER, causing disruption of the ER homeostasis and triggering the unfolded protein response pathway. Activation of this pathway is initially thought to restore cell homeostasis by attenuating protein translation, enhancing degradation of misfolded proteins, and increasing levels of ER chaperons and redox enzymes to increase folding capacity [51]. However, depending on the severity of the ER stress, the unfolded protein response can trigger pro-apoptotic pathways that shift the cellular fate towards cell death. In stressed or dying cells, calreticulin exposed on the cell surface serves as a phagocytic signal to DCs, resulting in the engulfment of cancer material by DCs and in tumor antigen presentation and anticancer cytotoxic T lymphocyte-specific responses [35,52].

Numerous pre-clinical studies have shown the potential of chemotherapy and/or radiotherapy to induce ICD by the upregulation of calreticulin [53,54,55,56]. However, only a limited number of studies have investigated this in patients. In 2012, Suzuki et al. analyzed the chemoradiotherapy (CRT)-induced expression of calreticulin in resected tumor samples from patients with esophageal squamous cell carcinoma (ESCC, N = 45) [37]. Immunohistochemical analysis was conducted to assess the intensity of membranous- and intracellular-located calreticulin in cancer cells. No differences in the intensity of calreticulin expression were observed between groups with and without pre-operative CRT, suggesting that calreticulin expression was not upregulated in response to CRT in ESCC samples. Of note, no differences in patient characteristics have been observed except for the grade of lymph node dissection. Additionally, the different stages of ESCC (stage I-IV) were equally distributed in both groups (with and without preoperative CRT), indicating the studied groups were biologically comparable.

In 2015, Garg et al. used publicly available datasets that comprised patients with various cancer types to assess calreticulin expression levels in pre-treatment tumor tissue samples and showed that at least a subset of patients of various cancer types have low tumoral expression of calreticulin [38]. This could imply that low endogenous levels of calreticulin could be a crucial cell autonomous factor compromising the effectiveness of anticancer treatments to induce an immunogenic response. In addition, the potential of calreticulin to serve as a predictive biomarker to predict the RT-induced ICD response was investigated in the non-small cell lung cancer (NSCLC) setting. It was shown that patients with NSCLC (treated with RT only, N = 23) with high calreticulin expression in the tumor (N = 8), tended to have significantly better overall survival (OS) compared with RT-treated patients with NSCLC with low calreticulin expression (N = 15). Moreover, experimental data suggested that low endogenous calreticulin levels are associated with low ecto-calreticulin exposure at the cell surface, which in turn attenuates the effectiveness of the DC response in antigen presentation and mounting an immune response. Interestingly, in RT-treated patients with NSCLC, tumoral calreticulin levels were positively correlated with tumor levels of phagocytosis-associated genes [38].

More recently, Murakami et al. investigated the potential immunogenic benefit of neoadjuvant CRT in patients with pancreatic cancer (N = 65), which is generally considered an immune-evasive malignancy [39]. Among these 65 patients, 51 underwent surgical resection after neoadjuvant CRT while 14 patients became ineligible for surgical resection due to disease progression during neoadjuvant CRT. Independent of the pathological tumor stage, high calreticulin levels were more often observed in tumor specimens of neoadjuvant treated patients (N = 33) compared to tumor specimens of treatment-naïve pancreatic cancer patients (N = 18). In addition, the absolute number of CD8+ and CD4+ tumor-infiltrating lymphocytes was significantly higher in the neoadjuvant treated patients than in the treatment-naïve group. Based on these data, the authors suggest that neoadjuvant CRT contributes to increased abundance of these DAMPs within the cell, which may play an important role in acquiring a favorable immune response for these patients. Nevertheless, in this retrospective study, it was not reported if there was a relationship between the immunologic tumor microenvironment and the clinical outcome (i.e., OS, PFS). Singh et al. performed a pilot study to investigate the contribution of high-dose RT in preconditioning patient tumors for effective anti-tumor responses [40]. Tumor cell surface expression of calreticulin was significantly increased in samples resected from patients with renal cell carcinoma treated with stereotactic body radiation therapy (SBRT) (N = 14) compared to control samples (N = 16).

#### 2.1.2. Heat-Shock Protein 70

ER-resident chaperones such as heat-shock protein 70 (Hsp70) also play a role in the immunogenicity of dying cancer cells. During ICD, these DAMPs are translocated to the cell membrane where they can interact with their respective counter-receptors CD40 and CD91 on DCs. Pre-clinical evidence revealed that the interaction of Hsp70 with the co-stimulatory molecule CD91 on DCs promoted the activation of CD8+ cytotoxic T cells via a predominantly Hsp70-dependent antigen cross-presentation process [57]. However, depending on its subcellular localization, Hsp70 can fulfill different functions in cancer cells, such as bypassing a cellular senescence program, supporting metastasis, interfering with tumor therapy, and it can be recognized by immune cells, causing initiation of signal transduction pathways [58]. In contrast to the suggested beneficial role of Hsp70 in DC-activation in pre-clinical studies, several human studies revealed that elevated Hsp70 levels are associated with a poor prognosis in a variety of tumor entities including osteosarcomas, squamous cell carcinoma of the lung, lower rectal cancer, and hematological diseases [59,60,61]. For example, Stangl et al. investigated the potential prognostic value of Hsp70 in patients with squamous cell carcinoma of the head and neck after CRT [41]. Increased levels of Hsp70 (N = 68), indicated by a high Hsp70 staining score, were associated with a lower OS, local progression (LPS), and distant metastases-free survival (MFS) compared with patients with a low Hsp70 tumor staining score (N = 77). Similarly, Qi and colleagues used Hsp70 as a biological marker, in combination with multimodal magnetic resonance imaging, to predict the treatment response in advanced cervical cancer treated with CRT (N = 101) [42]. Hsp70 levels were assessed in pre-treatment tissue biopsies and associated with treatment response-related outcome parameters. It was shown that the pre-treatment Hsp70 expression levels, tumor diameter, and tumor volume were lower in the complete response group than in the partial response group [42]. These data suggest that increased levels of Hsp70 are associated with a poor treatment response. Moreover, recurrence of the tumor was less often observed in patients with low Hsp70 expression. Based on these findings, the authors suggest that the Hsp70 protein, combined with multimodal MRI, may accurately predict the CRT efficacy in patients with advanced cervical cancer. Similar findings were also observed in a more recent study by Rothammer et al., who showed that breast cancer patients who developed a contralateral recurrence or metastases within the first 2 years after RT had significantly higher serum Hsp70 values at the end of RT up to 6 weeks after RT compared to patients who remained disease-free [43].

In contrast to the potential prognostic value of Hsp70 in RT-treated patients with cancer, Lämmer et al. recently showed that elevated cytosolic Hsp70 levels in tumor tissue of 60 glioblastoma patients were associated with a prolonged progression-free survival (PFS) and OS [44]. Contradictory results have been observed in a single-arm feasibility study from Singh et al. who analyzed the immunological impact of SBRT followed by cytoreductive nephrectomy in patients with metastatic renal cell carcinoma. No increase in the expression of surface molecule Hsp70 was observed on the tumor cells of patients treated with SBRT compared to control samples [40].

#### 2.1.3. High Mobility Group Box 1

High mobility group box 1, also called HMGB1, is a non-histone chromatin-binding protein localized in the nucleus, where it interacts with DNA and regulates transcription. When released from dying cells, extracellular HMGB1 has potent immunostimulatory effects by interacting with distinct pattern recognition receptors, such as toll-like receptor 2 (TLR2), TLR4, and RAGE [62,63,64]. RT-induced release of HMGB1 has been detected in vitro and in vivo [62,65,66]. However, there are only a limited number of clinical studies that have investigated whether or not HMGB1 can be used as a biomarker to monitor radiotherapy-induced ICD in patients. Similar to Hsp70, contradictory results have been reported. For example, Hongo et al. observed that high nuclear HMGB1 expression was associated with a poorer response to CRT in rectal cancer patients (high HMGB1 N = 52 vs. low HMGB1 N = 23), in terms of both the tumor reduction ratio and the post-CRT histological tumor regression grade [45]. Additionally, compared to patients with low nuclear HMGB1 expression in the tumor, patients with high HMGB1 expression were more often diagnosed with a moderately differentiated tumor type. In this setting, it is unclear if the increased HMGB1 levels are a reflection of the treatment response or if these levels are increased as a consequence of tumor-intrinsic features (i.e., tumor differentiation grade). Nevertheless, it should be noted that the authors focused on nuclear HMGB1 staining and not on the release of HMGB1 into the TME. In the nucleus, HMGB1 can act as a DNA chaperone to maintain nuclear homeostasis and loss of HMGB1 increases DNA damage by decreasing DNA repair efficiency in response to chemotherapy, RT, and oxidative stress [67]. Therefore, it is likely that increased HMGB1 levels in the nucleus may play a key role in RT-induced resistance mechanisms and are therefore not an appropriate biomarker for RT-induced ICD. In contrast to this study, Suzuki et al. evaluated the CRT-induced expression of HMGB1 within the TME in patients with esophageal squamous cell carcinoma [37]. The proportion of cancer cells that stained positive for HMGB1 in the TME was significantly more predominant in the group with preoperative CRT compared with the group without preoperative CRT. In addition, patients with high HMGB1 expressing tumors had a better OS compared to patients with low HMGB1 expressing tumors. The OS was further improved when patients were treated with neoadjuvant CRT, indicating that both tumor intrinsic and neoadjuvant CRT contribute to increased HMGB1 levels. Moreover, significantly increased levels of HMGB1 were observed in serum samples collected after CRT, whereas no detectable levels of HMGB1 were observed in serum samples collected before CRT (N = 14). Together, these data suggest that released HMGB1 may be a prognostic marker that can be used to monitor treatment response. In recent years, similar findings have been reported by other research groups. For example, Huang et al. showed that patients whose biopsy specimens demonstrated high cytosolic translocation of HMGB1 (N = 44) had no local recurrence and a lower distant metastases rate compared to those with a low level of cytoplasmic HMGB1 translocation (N = 45) [46]. All patients had biopsy-proven locally advanced rectal cancer and were treated with preoperative CRT. Moreover, the magnitude of cytosolic translocation of HMGB1 in the tumor cells before neoadjuvant CRT positively correlated with patient survival, implying cytosolic HMGB1 might be a predictive biomarker of treatment outcome. To investigate this hypothesis, Suwinski et al. evaluated the prognostic value of HMGB1 in serum samples of patients with NSCLC treated with either palliative or curative thoracic CRT [47]. All patients with stage I and II NSCLC were excluded from surgery due to comorbidities or lack of consent and were treated with radiotherapy alone (stereotactic treatment, 60 Gy in 3-5 fractions). HMGB1 levels were increased in the majority of patients, however, these circulating HMGB1 levels were not associated with a better OS, local-regional control (LRC), and MFS. Nevertheless, these analyses were performed in a heterogeneous group of patients with respect to the stage of the disease (stage I-IV), tumor pathology (i.e., squamous, adenocarcinoma), and general performance status. Although the tumor stage has a major influence on OS, the prognostic value of HMGB1 has not been analyzed within these tumor stage subgroups. To surpass the limitations of the heterogeneous patient population, the authors have analyzed the level of HMGB1 in patients that were treated with curative RT or CRT (N = 148). Again, serum levels of HMGB1 did not appear to be associated with OS. More recently, Bains et al. also aimed to investigate the hypothesis that HMGB1 in the patient’s circulation might act as a predictive biomarker for the ability of neoadjuvant CRT to induce ICD [48]. Nevertheless, only a modest increase in plasma HMGB1 was detected during neoadjuvant chemotherapy and sequential CRT compared to baseline. Interestingly, when the patients were categorized with respect to mutant or wild-type *KRAS* status, only the wild-type group revealed an increase in HMGB1 plasma levels upon neoadjuvant CRT. This is in correspondence to previous findings from Lal et al. who showed that colorectal cancers harboring mutant *KRAS* are associated with reduced infiltration of cytotoxic T-cells and suppression of the adaptive IFN-γ response [68].

### 2.2. Interferon Signaling

RT also boosts the anti-tumor response through induction of type I interferons [69]. RT-induced DNA strand breaks result in the aberrant appearance of DNA in the cytoplasm (e.g., micronuclei), triggering activation of the cGAS/STING/TBK1 signaling pathway, which ultimately results in the production of type I interferons (IFN α/β). Type I interferons play critical roles in activating both innate and adaptive immune responses. Although a low level of DNA damage is maintained in many cancer cells, potentially leading to chronic activation of the cGAS/STING/TBK1 pathway, the DNA damage induced by ionizing irradiation greatly exceeds this baseline and triggers much higher levels of type I interferons [13,70]. In addition, numerous studies showed that RT promoted the activation of anti-tumor T cells, which is dependent on type I interferon induction in the irradiated tumor [13,29,71].

A substantial body of evidence from immunocompetent murine models indicates that the combination of radiotherapy plus immune checkpoint blockade (ICB) with anti-cytotoxic T-lymphocyte-associated protein 4 (CTLA-4), anti-programmed cell death protein 1 (PD-1), or both, can enhance both direct and abscopal responses to radiotherapy. Acute induction of the STING pathway is of particular importance for the abscopal response and both tumor-intrinsic and exosomal-mediated mechanisms of acute IFN production probably contribute to this effect [69]. Concordant with studies in mice, Formenti et al. have recently reported the clinical outcomes and translational readouts of a trial of the anti-CTLA-4 inhibitor, ipilimumab, in combination with palliative radiotherapy in 39 patients with NSCLC [49]. A radiological response was detected in 18% of patients and 31% of patients experienced disease control. Simultaneously, serum IFNβ was significantly increased shortly after completion of RT in responders, whereas no significant increases occurred in patients with progressive disease [49].

### 2.3. Receptor-Interacting Protein

Apoptosis refers to an active form of regulated cell death in contrast to necrosis, which is regarded as a passive form of cell death. However, recent data suggest that necrosis can also be an alternate form of programmed cell death which activation may have important consequences, such as inducing an inflammatory response [72]. This form of regulated necrotic cell death is also called necroptosis. Over the past two decades, a plethora of molecules and processes have been characterized as initiators, modulators, or effectors of necroptosis, including, but not limited to, receptor-interacting protein 1 (RIP1; also known as RIPK1 and RIP3 (also known as RIPK3) [72]. Both kinases have been shown to be responsible for mediating necroptosis, and absence of RIP3 contributes to necroptosis resistance [73]. The TNF/TNF receptor 1 mediated signaling pathway is one of the most extensively studied models of necroptosis [74,75,76], which results in the activation of RIP1 by phosphorylation, which in turn activates RIP3 through its kinase activity resulting in the formation of the RIP1/RIP3 complex. This complex phosphorylates the mixed lineage kinase domain-like (MLKL) kinase, which is then transported into the nucleus and to the cell membrane, eventually triggering cellular membrane rupture and cell death [77]. Exposure of thyroid and adrenocortical carcinoma cell lines to increasing doses of RT in the presence of necroptosis inhibitor Nec-1 resulted in significantly increased cell survival compared to controls [78]. Wang et al. investigated if RIP3 and necroptosis were associated with the clinical outcomes of eligible early-stage NSCLC patients treated with SBRT (N = 21) [50]. High expression of RIP3 in the tumor specimen (N = 8) was associated with improved local tumor control and PFS, suggesting RIP3 may serve as a useful biomarker to predict favorable response to SBRT.

## 3. Discussion

In this review, we have summarized the available literature on potential biomarkers of RT-induced ICD that have been studied in the clinical setting so far. Notably, most studies focused on the expression and release of DAMPs, including calreticulin, HMGB1, and Hsp70. These are molecules that are exposed or released by dying cells and that are sensed by innate immune cells, hence they can promote the activation and maturation of these cells in order to elicit an adaptive immune response. Although numerous studies have reported a potential predictive or prognostic role for these ICD-associated molecules, contradictory results have also been reported in relation to treatment response and outcome. Furthermore, assessment of the clinical applicability of these biomarkers is currently still hampered by the relatively small size of these studies, the inclusion of heterogeneous patient populations (i.e., disease stage), and differences in treatment (i.e., RT dose). Here, we will discuss the clinical relevance of these findings and important aspects that need to be considered in studying biomarkers of RT-induced ICD.

The synergy of RT and the immune system is currently receiving significant attention as numerous studies have shown that radiotherapy can induce strong anti-tumor immune responses [79]. As such, combination treatment regimens involving RT and immunotherapy are being developed and clinically tested to optimize these synergistic effects and improve the clinical outcome of cancer patients. For example, recent results of the randomized, placebo-controlled, phase III randomized PACIFIC trial revealed an improved OS at 4 years which was increased from 36.3% to 49.6% with the addition of durvalumab (anti-PD-L1), given for 12 months after concurrent CRT [80,81,82,83]. Nowadays, adjuvant durvalumab has become standard of care for patients with locally advanced (stage III) NSCLC treated with concurrent CRT that did not progress after CRT, and have a good performance status, plus have recovered from their CRT induced acute toxicity. However, it should be noted even with the addition of durvalumab to concurrent CRT, only half of the patients are alive at 4 years, and more have progressed already, either locally or distant [83]. To date, it remains a challenge to identify the patients that will benefit from these combination treatments. A key aspect that determines whether or not patients will benefit from these immunomodulatory treatments is the immunological landscape of the tumor and the host. Nevertheless, despite the many efforts to improve our understanding of the fundamental processes underlying RT-induced ICD and to identify immunogenic factors that are released by irradiated tumor cells, there are currently no validated biomarkers available for the routine monitoring of these RT-induced immunomodulatory effects [84].

Biomarkers are defined as characteristics that can be evaluated and measured as indicators of normal biological processes, pathogenesis, or response to therapy [85]. Cancer biomarkers may be diagnostic, prognostic, predictive, or used to monitor treatment responses. Whereas prognostic biomarkers provide information about a patient’s overall cancer outcome, irrespective of therapy, predictive biomarkers indicate the probability of a patient gaining a therapeutic benefit from a specific treatment. To be able to identify individuals in which RT can induce an immunogenic response, we need to identify biomarkers that can predict response to RT before starting a treatment or biomarkers that can help clinicians to assess the RT-induced immune response during treatment. In the current review, we noted that potential biomarkers of RT-induced ICD have predominantly been analyzed in surgically obtained tumor samples from cancer patients. These samples have been collected either before or after RT treatment, which has important implications for the interpretation; whereas neoadjuvant RT followed by surgery allows the study of the local presence of ICD biomarkers that are expressed in response to therapy, the collection of tumor tissue before adjuvant RT only allows to assess the predictive value of the endogenous levels of these ICD biomarkers.

The expression of Hsp70 has predominantly been assessed in pre-treatment tumor tissue samples and has been associated with treatment response and outcome. Increasing evidence suggests that Hsp70 exerts dual roles as increased levels of Hsp70 have been associated with a lower OS and PFS [59,60,61], but Lämmer et al. identified Hsp70 as a prognostic marker that was associated with a favorable outcome [44]. These contradictory results may be explained given the emerging knowledge on the diversity of Hsp70 functions in cancer [86,87,88]. Although Hsp70 may have a prognostic or predictive role in cancer patients treated with CRT, it has not been investigated if increased expression of Hsp70 is associated with the recruitment and activation of DCs in the TME. During ICD, Hsp70 is exposed on the cell membrane of dying cells and ultimately released in the TME [9]. Hsp70 presented on the cell surface allows for the interaction of these proteins with their respective receptors, CD40 and CD91, on DCs [89]. Pre-clinical studies have shown that Hsp70 can stimulate DC maturation by promoting the upregulation of CD86 and CD40 on DCs and, through these co-stimulatory signals, augments CD8+ cytotoxic T lymphocyte-mediated immune responses [90,91]. Given the potential local immunological roles of Hsp70, it is recommended to assess RT-induced Hsp70 expression levels on the surface of tumor cells in tumor tissue samples. Additionally, further investigation is needed to determine if RT-induced upregulation of Hsp70 is associated with the recruitment and activation of DCs in tumor biopsies from cancer patients.

A potential link between the upregulation of ICD-associated biomarkers and the recruitment of DCs to the TME in cancer patients has only been reported for calreticulin. For example, Fucikova et al. showed that patients with NSCLC, who presented with high calreticulin expression in the tumor, also exhibited increased numbers of infiltrated DCs in the TME [92]. In addition, irrespective of RT treatment, calreticulin expression has also been associated with an improved OS [92,93,94,95,96]. To establish the use of calreticulin as a potential predictive biomarker, future studies are warranted. First, the expression of calreticulin should be assessed in larger and well-defined, homogeneous (i.e., tumor type, tumor stage, treatment) patient populations. This is required to identify treatment-associated upregulation of calreticulin. To assess whether or not RT-induced expression of calreticulin results in the initiation of an anti-tumor immune response, it is also key to assess the presence of immune cell subtypes (e.g., DCs, CD8+, CD4+, Tregs) in the TME. This facilitates the evaluation of a possible association between the RT-induced upregulation of calreticulin and the recruitment of immune cell subsets that are in favor of targeting the tumor.

HMGB1 may be another biomarker that can be used to predict if RT can induce ICD [37,45,46,48]. However, it is important to mention that among these studies, HMGB1 expression levels have been analyzed in both tumor and blood samples. In the tumor tissue samples, HMGB1 expression levels have been analyzed at different locations (i.e., nuclear, cytoplasmic, extracellular), which may be associated with a different (functional) outcome. HMGB1 is also released in other situations than in ICD and together with the lack of a standardized measurement, it is therefore unlikely that HMBG1 on its own will be suitable as a biomarker for ICD.

In contrast to the assessment of ICD-associated biomarkers in tumor tissue samples, circulating biomarkers can act as surrogates for local immune mechanisms in the TME, allowing for a more comprehensive evaluation of the host’s immune status and longitudinal testing [97]. Additionally, blood-based biomarkers are easier to obtain compared to tissue biopsies and can be collected at multiple time points, the latter being a prerequisite to gain insight on the (temporal) RT-induced immunomodulatory changes. Given the fact that treatment-induced ICD is associated with the expression and release of molecules that can elicit a systemic immunogenic response, it is very likely that blood contains molecules that may be reflective of this RT-induced immune response. However, so far, only a limited number of studies have assessed the expression of potential biomarkers of ICD in the blood of cancer patients [49]. In addition, most studies have been performed with small and heterogeneous patient populations. Large-scale prospective studies with well-defined patient populations (i.e., disease stage, treatment) are required in obtaining robust results. Additionally, the use of standardized methodological approaches is key for the interpretation of results of multiple prospective studies.

The adequate selection of the most appropriate sample type and the collection and handling thereof is an integral component of biomarker research. In the context of RT-induced ICD, it should be recognized that RT is generally considered to be a local treatment. However, local irradiation of the tumor cells will result in both local and systemic immunogenic responses. Although accumulating preclinical evidence indicates that RT-induced ICD is accompanied by the exposure, active secretion, or passive release of numerous DAMPs [5], it is striking that in the majority of studies only one ICD-associated molecule is studied. A single biomarker can generally not be used to monitor treatment-induced immune responses, and therefore, multiple biomarkers should be studied simultaneously at different time points. Moreover, increasing evidence reveals that RT can exert both immunostimulatory and immunosuppressive effects [98]. Recently, Hayashi et al. also demonstrated that the induction of ICD is not only associated with the ectopic expression and release of immuno-stimulatory DAMPs but is also associated with the release of immunoinhibitory DAMPs that may counterpoise the adjuvanticity of the immunostimulatory DAMPs [99]. It is therefore postulated that an intricate balance between immunostimulatory and inhibitory DAMPs and cytokines could determine the outcome of treatment-induced ICD. This further supports the assessment of multiple biomarkers and links the expression levels to the net functional immunological consequences. Furthermore, a comprehensive view on RT-induced ICD should ideally be obtained by studying both tumor and blood specimens. This allows to identify the expression levels of multiple ICD-associated molecules both locally and systemically and to perform correlation analyses. These studies will highly contribute to broadening our knowledge on treatment-induced ICD and the induction of an anti-tumor immune response in cancer patients. Ultimately, the identification and validation of a panel of immunological biomarkers may help clinicians to monitor the treatment response and to select the most optimal treatment combinations.

## 4. Conclusions

The potential prognostic and predictive value of biomarkers of RT-induced ICD has only been investigated in a limited number of clinical studies. Circulating biomarkers are promising in the field of RT-induced ICD as blood-based biomarkers can reflect tumoral characteristics, provide information on the host’s immune status, can be evaluated longitudinally, and multiple biomarkers can be assessed simultaneously without sample limitations. Furthermore, systematic evaluation of ICD-associated biomarkers in both tumor tissue and blood samples may offer a more comprehensive understanding of treatment-induced immune response locally and systemically. Prospective clinical trials with well-defined patient populations and treatment regimens are urgently needed for the identification of ICD-associated biomarkers. In the future, these biomarkers may enable to define synergistic treatment combinations for the optimal priming of the immune system.

## Figures and Tables

**Table 1:** Potential biomarkers of RT-induced immunogenic cell death in cancer patients.

ICD-Biomarker	Treatment	Cancer Type	Sample	Nr Treated Patients	Nr Control Subjects	Treatment-Induced Effect(s) on ICD-Biomarkers	Associated with Immune Response	Associated with Outcome	Author (Year)	Ref
Calreticulin	CRT (Pre-operative)	ESCC	Tumor tissue	45	43	No upregulation of calreticulin	ND	ND	Suzuki et al. (2012)	[37]
	RT (Pre-operative)	NSCLC	Publicly available dataset	23	227	↑ Calreticulin	↑ phagocytosis- related genes	↑OS	Garg et al. (2015)	[38]
	CRT (Pre-operative)	Pancreatic cancer	Tumor tissue	51	33	↑ Calreticulin	Not associated with pre-operative TILs-, leukocyte-, lymphocyte-, and neutrophil-count	ND	Murakami et al. (2017)	[39]
	SBRT (Pre-operative)	mRCC	Tumor tissue	14	16	↑ Calreticulin	ND	ND	Singh et al. (2017)	[40]
Hsp70	SBRT (Pre-operative)	mRCC	Tumor tissue	14	16	No upregulation Hsp70	ND	ND	Singh et al. (2017)	[40]
	CRT (Pre-operative)	Pancreatic cancer	Tumor tissue	51	33	↑ mHsp70	Not associated with pre-operative TILs-, leukocyte-, lymphocyte-, and neutrophil-count	ND	Murakami et al. (2017)	[39]
	CRT (Post-operative)	SCCHN	Tumor tissue	145	-	↑ Hsp70	ND	↓ OS, LPFS, DMFS	Stangl et al. (2018)	[41]
	CRT (Post-operative)	Cervical cancer	Tumor tissue	101	-	↓ Hsp70	ND	↑ CR	Qi et al. (2018)	[42]
	RT (No surgery)	Breast cancer	Blood	35	-	↑ sHsp70	ND	↑ Tumor recurrence or metastases	Rothammer et al. (2019)	[43]
	CRT (Post-operative)	GBM	Tumor tissue	60	-	↑ cytosolic Hsp70	ND	↑ PFS, OS	Lämmer et al. (2019)	[44]
HMGB1	CRT (Pre-operative)	ESCC	Tumor tissue	45	43	↑ HMGB1	↑ CD8+ TILs (tendency)	ND	Suzuki et al. (2012)	[37]
			Blood	14	-	↑ HMGB1	Associated with antigen-specific T-cell response	ND		
	CRT (Pre-operative)	Rectal cancer	Tumor tissue	75	-	↑ HMGB1	ND	↓ Tumor regression	Hongo et al. (2015)	[45]
	CRT (Pre-operative)	Rectal cancer	Tumor tissue	89	-	↑ HMGB1	ND	↑ Tumor regression	Huang et al. (2018)	[46]
	RT (No surgery)	NSCLC	Blood	206	-	↑ HMGB1	ND	Not associated with 3-yr OS, LRC, MFS	Suwinski et al. (2019)	[47]
	CRT (Pre-operative)	Rectal cancer	Blood	50	-	↑ HMGB1	ND	↑ MFS	Bains et al. (2020)	[48]
Interferon type I	RT, Ipilimumab (No surgery)	NSCLC	Blood	39		↑IFN-β	ND	↑ Treatment response	Formenti et al. (2018)	[49]
RIP3	RT (Post-operative)	NSCLC	Tumor tissue	21		↑RIP3	ND	↑ LRC, PFS	Wang et al. (2018)	[50]

CR, complete response; CRT, chemoradiotherapy; DMFS; distant metastases free survival; ESCC, esophageal squamous cell carcinoma; GBM, glioblastoma; LPFS, local progression free survival; LRC, locoregional control; MFS, metastases-free survival; mRCC, metastatic renal cell carcinoma; nr, number; NSCLC, non-small cell lung cancer; OS, overall survival; PFS, progression free survival; RT, radiotherapy; SBRT, stereotactic body radiation therapy; SCCHN, squamous cell carcinoma of the head and neck; sHsp70, soluble heat shock protein 70; TILs, tumor-infiltrating lymphocytes; TMZ, temozolomide; TRG, tumor regression grade. ND = not determined.

## Data Availability

Not applicable.
